# Desert Environments Facilitate Unique Evolution of Biosynthetic Potential in *Streptomyces*

**DOI:** 10.3390/molecules26030588

**Published:** 2021-01-22

**Authors:** Kunjukrishnan Kamalakshi Sivakala, Karina Gutiérrez-García, Polpass Arul Jose, Thangadurai Thinesh, Rangasamy Anandham, Francisco Barona-Gómez, Natesan Sivakumar

**Affiliations:** 1 School of Biotechnology, Madurai Kamaraj University, Madurai 625021, India; sivakalaarul@gmail.com; 2 Robert H. Smith Faculty of Agriculture, Food and Environment, Hebrew University of Jerusalem, Rehovot 7610001, Israel; 3 Evolution of Metabolic Diversity Laboratory, Unidad de Genómica Avanzada (Langebio), Cinvestav-IPN, Km 9.6 Libramiento Norte, Carretera Irapuato-León, CP 36821 Irapuato, Guanajuato, Mexico; kgutierrez@carnegiescience.edu (K.G.-G.); francisco.barona@cinvestav.mx (F.B.-G.); 4 Department of Embryology, Carnegie Institution for Science, Baltimore, MD 21218, USA; 5 Department of Agricultural Microbiology, Agricultural College and Research Institute, Tamil Nadu Agricultural University, Madurai 625104, India; anandham@tnau.ac.in; 6 Department of Microbiology, School of Life Sciences, Pondicherry University, Puducherry 605014, India; thina.sathesh@gmail.com

**Keywords:** actinobacteria, genome mining, metabolomics, secondary metabolites, natural products, arid environment

## Abstract

Searching for new bioactive metabolites from the bacterial genus *Streptomyces* is a challenging task. Combined genomic tools and metabolomic screening of *Streptomyces* spp. native to extreme environments could be a promising strategy to discover novel compounds. While *Streptomyces* of desertic origin have been proposed as a source of new metabolites, their genome mining, phylogenetic analysis, and metabolite profiles to date are scarcely documented. Here, we hypothesized that *Streptomyces* species of desert environments have evolved with unique biosynthetic potential. To test this, along with an extensive characterization of biosynthetic potential of a desert isolate *Streptomyces* sp. SAJ15, we profiled phylogenetic relationships among the closest and previously reported *Streptomyces* of desert origin. Results revealed that *Streptomyces* strains of desert origin are closer to each other and relatively distinct from *Streptomyces* of other environments. The draft genome of strain SAJ15 was 8.2 Mb in size, which had 6972 predicted genes including 3097 genes encoding hypothetical proteins. Successive genome mining and phylogenetic analysis revealed the presence of putative novel biosynthetic gene clusters (BGCs) with low incidence in another *Streptomyces*. In addition, high-resolution metabolite profiling indicated the production of arylpolyene, terpenoid, and macrolide compounds in an optimized medium by strain SAJ15. The relative abundance of different BGCs in arid *Streptomyces* differed from the non-arid counterparts. Collectively, the results suggested a distinct evolution of desert *Streptomyces* with a unique biosynthetic potential.

## 1. Introduction

*Streptomyces* spp. are aerobic Gram-positive bacteria well-known for their unprecedented potential to produce commercially important natural compounds. Discovery of streptomycin, the first commercially successful antibiotic from *Streptomyces*, inspired large-scale isolation of these filamentous bacteria from different environments and resulted in the discovery of a range of antibiotics, immunosuppressives, antihelminthics, and anticancer drugs [1,2,3,4]. Consequently, *Streptomyces* spp. are recognized as major players in the field of natural product discovery. Furthermore, the discovery of new natural products is quite challenging due to the increasing re-isolation of known metabolites.

Unusual environments previously unexplored for novel *Streptomyces* are considered as potential sources for natural compound discovery. Notably, arid soils [5,6], hypersaline soils [7], red soils [8], intertidal sediments [9], and deep-sea sediments [10] have recently been recognized as sources of novel *Streptomyces* spp. in recent years. In arid environments, microorganisms encounter extreme desiccation condition often accompanied by extreme temperature levels, intense radiation, low levels of nutrients, low water activity, and high salinity [5,11,12]. Distilled by multiple life-history traits, ecologically coherent, oligotrophic microbial communities in the arid ecosystem serve as a rich source of novel functions [13]. The occurrence of novel *Streptomyces* spp. suitable for natural product discovery in desert environments has been revealed by some studies [6,12,14,15]. As a notable example, a recent study suggested endemicity of culturable Actinobacteria, especially *Streptomyces* in an extremely oligotrophic desert oasis [15]. In this context, our recent bioprospecting study focused on Actinobacteria inhabiting arid soil from the Thar Desert in India, and yielded isolate *Streptomyces* sp. SAJ15 with promising potential for the discovery of novel natural products. The Thar Desert is biogeographically at the easternmost edge of the Saharan-Arabian desert zone, which is known for its hyperaridity. Moreover, recent culture-dependent and culture-independent surveys of bacteria in the Thar Desert soils indicated the presence of potentially diverse *Streptomyces* spp. in this desert [6,12,14].

In recent years, the discovery of novel compounds from *Streptomyces* spp. has reinvigorated research into the biosynthetic potential hidden in their genomes [16,17,18]. The renowned importance of *Streptomyces* as a source of natural products, and the advancements of next-generation sequencing technologies, have favored genome sequencing of several *Streptomyces* spp. [19,20]. Mining of these genomes is an effective way to explore novel biosynthetic gene clusters (BGCs) with the hope of discovering novel compounds [20]. Accordingly, several natural compounds have been discovered from *Streptomyces* isolated from terrestrial, mangrove, and marine environments [16,21,22,23,24]. Genome mining of desert *Streptomyces* has unveiled new desferrioxamine traits in several *Streptomyces* isolated from the Chihuahuan desert of Mexico [25]. The *Streptomyces leeuwenhoekii* strains native to the hyperarid Atacama Desert in Northern Chile [26,27] produces several bioactive compounds [27,28,29], and has been progressively delineated as a “gifted” actinomycete after genome mining revealed the presence of several specialized biosynthetic gene clusters in this organism [30]. Moreover, *Actinobacteria* dominate in the arid soils of Antarctica, where metabolic adaptations influence survival under harsh conditions [31].

Thus, isolating novel *Streptomyces* spp. from underexplored environments and exploring their genetic potential can be considered as having substantial value in natural product research. In this study, we performed genome sequencing of the putative novel strain SAJ15 and mined the assembled draft genome for BGCs, including polyketides and non-ribosomal peptides biosynthetic systems. In addition, to assess readily accessible metabolites in laboratory conditions, we studied the secondary metabolites profile of this strain, in an ideal culture medium, by HR-LC-MS analysis. Our results reinforce the view that *Streptomyces* spp. from deserts present unique biosynthetic features with great promise for the discovery of novel natural products.

## 2. Results and Discussion

### 2.1. Taxonomic Identification and Phylogenetic Position of Strain SAJ15

**Figure 1:**
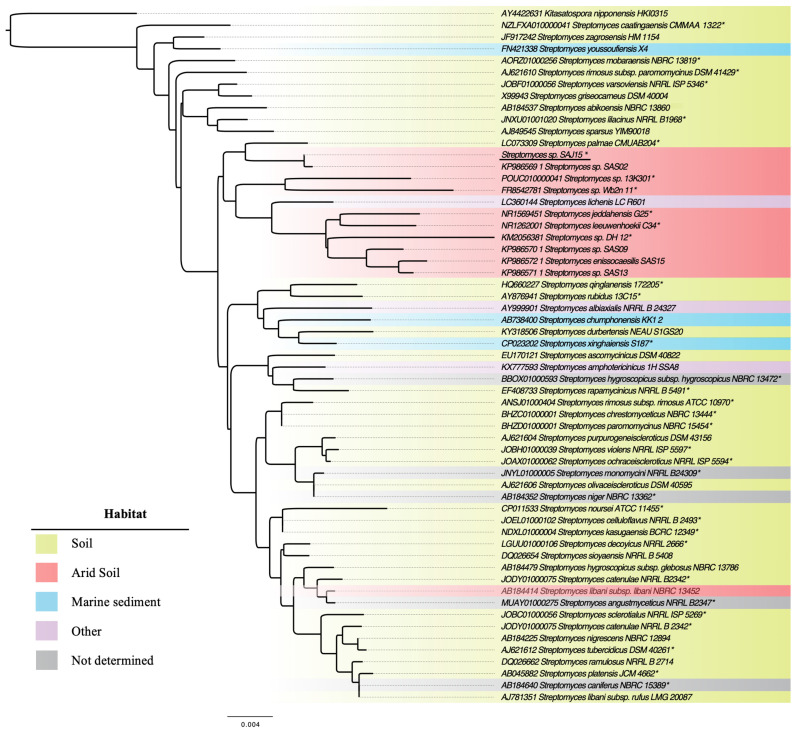
*Streptomyces* phylogenetic tree based on 16S rRNA gene sequence. SAJ15 forms a monophyletic clade with the desert isolate SAS02. Most of the *Streptomyces* isolated from arid-soil form a clade taxonomically near to SAJ15. *Kitasatospora nipponensis* HKI 0315 was used as an outgroup. Habitats for each *Streptomyces* isolates are indicated with colored bullets. Genome assembly availability is marked with an asterisk.

Taxonomic identification of the isolate SAJ15 was performed based on its 16S rRNA gene sequence using EzBioCloud’s Identify service (https://www.ezbiocloud.net/). SAJ15 is a member of the genus *Streptomyces*, showing the highest similarity score (98.69%) with *S. palmae* CMU-AB204^T^ [32]. To gain insights into the taxonomic position of strain SAJ15 within the genus *Streptomyces*, and the relationship with other arid-soil derived *Streptomyces*, we reconstructed a 16S rRNA phylogenetic tree using 58 gene sequences (Table S1). In the resulting tree (Figure 1), SAJ15 forms a monophyletic clade with *Streptomyces* sp. SAS02 and *S. palmae* CMU-AB204^T^. The closest strain SAS02 has previously been isolated from desert soil and found to have an untapped biosynthetic potential to produce novel metabolites based on molecular screenings and metabolic profiling [6]. Importantly, most of the *Streptomyces* strains isolated from arid-soil form a monophyletic clade taxonomically near to SAJ15, suggesting a speciation event according to a specific ecological niche [33,34].

### 2.2. Description of Draft Genome of Streptomyces sp. SAJ15

Genome sequencing of strain SAJ15 was performed using Illumina 101 bases PE sequencing, resulting in 78.49 million reads. Overall data quality was very good with 96.74% (75.93 million) high-quality filtered reads out of 78.49 million total reads. The quality-filtered reads were primarily assembled into 109 contigs with an average length of 0.07 Mb. The contigs were further assembled into 70 scaffolds covering 8.2 Mb genome. The scaffolded de novo genome assembly was validated and quality-checked by evaluating the read mapping, alignments, depth, and coverage. Reads mapped to the assembly concordantly and discordantly were 29,534,132 (29.53 million) and 7,300,330 (7.3 million), respectively. Overall alignment rate was 99.07%. Average depth and coverage were 1380.3x and 99.43% at scaffold level. These measures are comparable to the valid de novo genome assemblies of other *Streptomyces* species [35]. Output size of draft genome size was 8.2 Mb with a high GC content of 72.6%. The resulted draft genome assembly had good accuracy, short-range contiguity, long-range connectivity, and coverage of the genome. This (8.2 Mb) larger genome size is closer to that (8.6 Mb) of model strain *Streptomyces coelicolor* A3 [36] and equal to the genome of *Streptomyces* sp. Wb2n-1132—native to a desert environment [37]. Sequence data were deposited in the GenBank under accession number NISY00000000.

Draft genome assembly of *Streptomyces* sp. SAJ15 was found to have 6972 predicted genes within 45 scaffolds. Out of the 6972 predicted genes, 6896 were protein-coding genes and 76 non-protein coding genes. Moreover, a total of 3875 genes were predicted to encode characterized proteins, whereas the remaining 3097 (44.9%) were hypothetical proteins. Similarly, genome of desert isolate *Streptomyces* sp. Wb2n-11 has been reported to have 3368 hypothetical proteins [37].

### 2.3. Genome Mining of Novel Biosynthetic Gene Clusters in SAJ15

In order to comprehensively investigate possible novel BGCs encoding specialized compounds, the genome assembly of *Streptomyces* sp. SAJ15 was mined by antiSMASH 5.0 pipeline [38]. Our analyses revealed the presence of 43 BGCs within the *Streptomyces* sp. SAJ15 genome. Considering completeness, a total of 36 BGCs were considered for further analysis (Supplementary Information, Table S2). These BGCs were classified as two non-ribosomal peptide synthetases (NRPS), four type I polyketide synthases (T1-PKS), three type II polyketide synthases (T2-PKS), two type III polyketide synthases (T3-PKS), and eight hybrid PKS and NRPS BGCs. Additionally, lassopeptides, bacteriocin, lantipeptide, ladderane, ectoine, and terpene BGCs were also found in the genome of strain SAJ15. Only two BGCs were identified as responsible for the synthesis of known compounds, piericidin and ectoine [39,40,41,42]. In addition, ten BGCs showed sequence homology above 50% with respect to BGCs deposited within the MIBiG database (https://mibig.secondarymetabolites.org/index.html), which contains only BGCs with robust experimental evidence. Seventeen BGCs within the SAJ15 genome assembly showed low sequence homology (between 2 and 42%), while ten BGCs showed null sequence homology with bacterial BGCs reported previously. Out of these, 27 BGCs were unique (with sequence identity cutoff <20%), suggesting novel BGCs could encode new specialized compounds.

A total of 36 non-redundant and well-defined BGCs from SAJ15 were used to complement the *Streptomyces* BGCs database constructed with 6556 BGCs detected by antiSMASH 5.0. This included 191 public *Streptomyces* genome assemblies isolated from different environments, including all the genomes available from those strains isolated from arid soil (n = 5), genome assemblies from the 16S rRNA tree (n = 29), and the MIBiG database (1802 BGCs) (Table S3). To explore the diversity of the BGCs across the *Streptomyces*-BGCs dataset, we constructed the BGC sequence similarity networks using BiG-SCAPE [43]. The *Streptomyces* BGCs were classified into eight BiG-SCAPE families (GCFs) including (1) others, (2) terpenes, (3) NRPs, (4) RiPPs, (5) PKSI, (6) PKS-other, (7) PKS-NRPs hybrid, and (8) saccharides. Others and terpenes were the two GCFs most represented with the highest number of BGCs. Few BGCs from arid-soil present in big gene cluster clans (GCC) composed by more than 100 clusters were found, representing BGCs highly conserved and distributed in *Streptomyces*. Nevertheless, most of the BGCs from arid-soil were found in GCC with less than six *Streptomyces*-BGCs, with none of these BGCs belonging to BGCs previously characterized according to the MiBIG database (Supplementary Figures S1–S7, Table S3).

**Figure 2:**
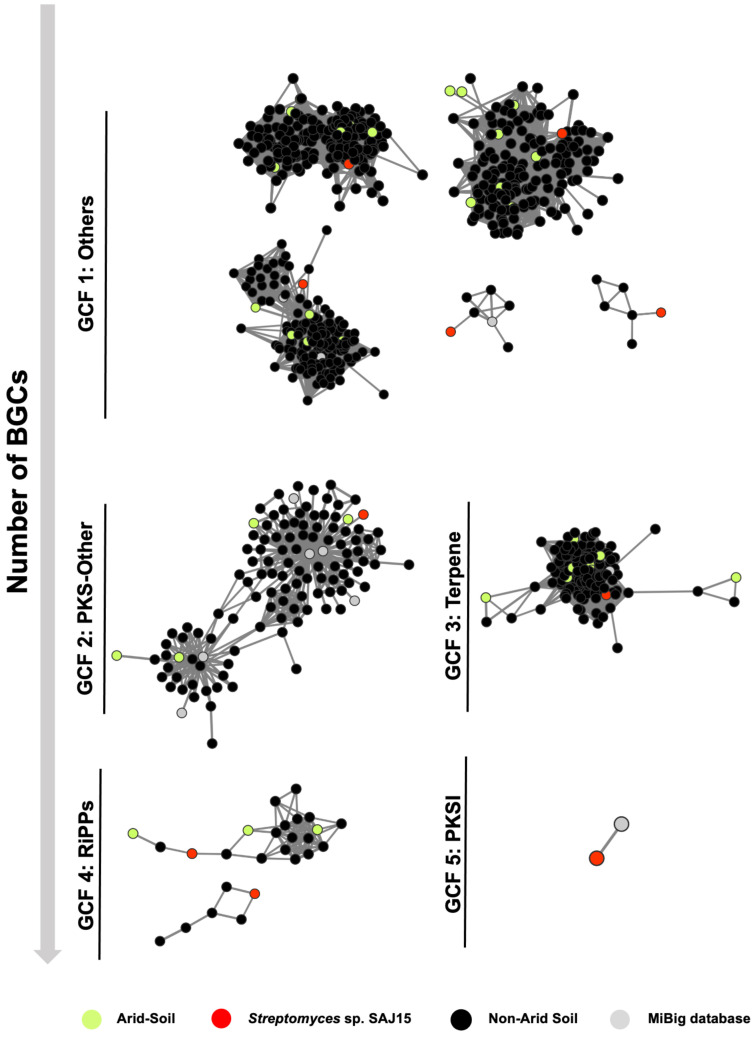
Simplified version of biosynthetic gene cluster (BGC) sequence similarity networks of BGCs from SAJ15 using 179 *Streptomyces* genomes. In total, 6556 *Streptomyces*-BGCs were classified into BiG-SCAPE families (GCFs). Only ten BGCs from SAJ15 were found in the five GCFs. Most of the BGCs from arid-soil were found in gene cluster clans (GCC) with less than six *Streptomyces*-BGCs, suggesting poor distribution and specificity within the genus *Streptomyces* (Supplementary Figures S1–S7).

Figure 2 represents the simplified version of the BGC sequence similarity networks of each SAJ15-GCF. Only ten BGCs from SAJ15 were found in five GCFs including (1) others, (2) PKSI, (3) PKS-other, (4) RiPPs, and (5) terpenes. Moreover, most of the GCC groups are composed by two or more BGCs, suggesting that those BGCs encode biosynthesis of highly similar or identical molecules with respect to other *Streptomyces* isolated from different environments. Additionally, 26 BGCs from SAJ15 were identified as singletons; this means BGCs that are not included in sequence similarity networks due to having a distance lower than the cutoff distance specified (Default C = 0.3). (Table S2).

These results suggest that most of the BGCs from arid-soil are poorly distributed into the previously known members of the genus *Streptomyces*.

**Figure 3:**
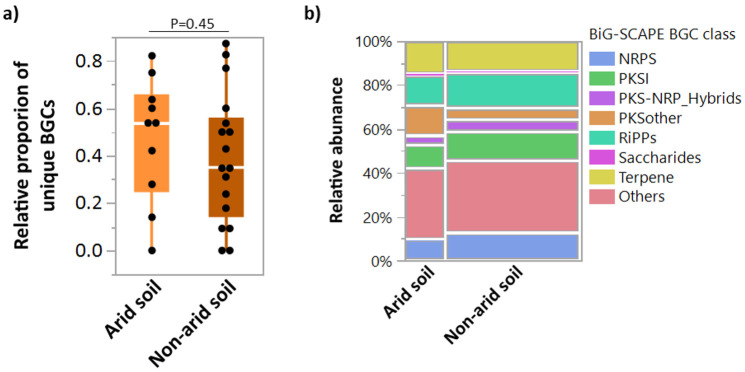
Relative abundance and BGC class distribution in arid soil and non-arid *Streptomyces*. The BGCs database was constructed with 6556 BGCs detected by atiSMASH 5.0 using 191 public *Streptomyces* genome assemblies isolated from different environments. Box plot shows relative proportion of unique BCGs in arid-soil derived *Streptomyces* in comparison to non-arid counterparts (**a**). Mosaic plot showing relative abundance of different BGC classes in the genome assemblies of arid and non-arid-based *Streptomyces* (**b**).

Bacterial strains isolated from harsh environments, such as arid soils, have been described as a rich source of novel specialized compounds [5]. To explore if arid environments harbor a higher number of unique BGCs in *Streptomyces*, their relative proportion was analyzed among the genome assemblies of *Streptomyces* reported from arid and non-arid environments (Figure 3). The relative proportion of unique BGCs is similar (Welch’s test, P = 0.45) in both arid- and non-arid-based *Streptomyces* (Figure 3a); however, relative abundance of different BGC classes differed in arid *Streptomyces* from non-arid counterparts (Likelihood-ratio test, χ^2^ = 16.68, P = 0.0195). Notably, occurrence of NRPS declined, with the notable increase in “PKS-Other” and terpene BGCs (Figure 3b). The specific enrichment of certain BCGs is in line with the growing observations on endemicity of microbial communities and functionalities in the desert ecosystems [13,15].

### 2.4. HPLC(PDA)-Guided Selection of Fermentation Medium

Secondary metabolite production is not a static property of microorganisms, and it can be significantly affected by constituents of culture media [44,45]. Notably, sources of nitrogen, such as amino acids, protein extracts, and inorganic nitrogen sources have been found to influence the production of secondary metabolites by *Streptomyces* species [44,46]. Therefore, *Streptomyces* sp. SAJ15 was cultivated in five different production media, and their biomass and secondary metabolites were quantified in triplicate (Supplementary Information Table S4).

**Figure 4:**
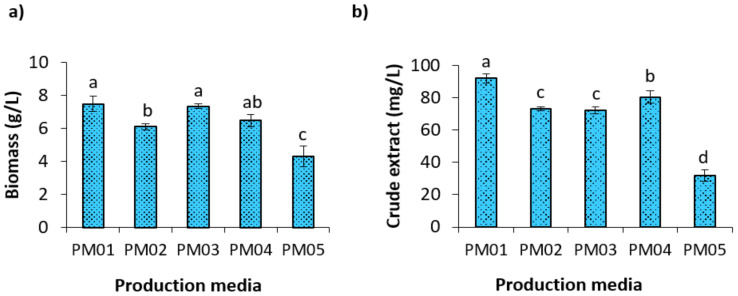
Effect of different production media (PM01 to 5, Table S4) on growth of (**a**) secondary metabolite production (**b**) in *Streptomyces* sp. SAJ15. Growth was measured as dry mycelial biomass in g L^−1^. Crude extracts were obtained using the standard liquid–liquid extraction method and expressed in mg L^−1^ (Mean ± SD). Different lowercase letters across denote statistically significant differences (*p* < 0.05 level).

All the production media, PM01 to PM05, used in the study favored good growth (Figure 4a) and varied levels of secondary metabolite production (Figure 4b). Notably, both PM01 and PM03 favored the maximum growth, with biomass weights of 7.48 ± 0.45 and 7.34 ± 0.13 g/L, respectively. However, the quantity of crude extract was high in PM01 that contained soluble starch as a carbon source, NH_4_SO_4_ as the major nitrogen source, and yeast extract as supplementary nitrogen. This result suggested that supplementation of a trace of yeast extract with inorganic nitrogen is best for secondary metabolite production by *Streptomyces* sp. SAJ15. Similar to this, yeast extract had a great influence on the production of secondary metabolites, for instance, actinorhodin by *S. coelicolor* [47]. In the case of PM05, glucose favored the growth of SAJ15, but showed a negative effect on the production of secondary metabolites, as evidenced by a low quantity of crude compound (Figure 4).

In order to detect the relative number of compounds produced by *Streptomyces* sp. SAJ15 in each medium, the crude-extracts were subjected to chemical profiling using HPLC-PDA analysis. Retention time of major peaks (Supplementary Information Figure S8) was counted and compared among the different production media. A maximum of 11 major peaks, which represent different compounds, were detected in the crude extract CE15-PM01, followed by CE15-PM02, CE15-PM03, and CE15-PM04, with five peaks. CE15-PM05 extracted from PM05 was found to have a smaller number of compounds with a single peak. Notably, compounds with a retention time (RT) of 5.21, 9.68, 14.8, and 18.2 were detected only in CE15-PM01. These results suggested that PM01 is the most suitable medium for the production of secondary metabolites by strain SAJ15. This is consistent with a recent study which revealed that the metabolic profile of *Streptomyces* sp. strain C34, isolated from the Chilean hyper-arid Atacama Desert soil, is dependent on the culture media used for its growth [29].

### 2.5. Secondary Metabolites Profiling Using HR-LC-MS

CE15-PM01, crude-extract obtained from the fermentation broth of *Streptomyces* sp. SAJ15, was subjected to high-resolution UHPLC-MS-Q-TOF analysis and a database search for metabolite identification (Supplementary information Figure S8). RT and the unique molecular mass of putative compounds are summarized in the Supplementary Information, Table S5. The molecular mass of the putatively novel compounds was in a range between 151.0384 and 489.311. Although not quantitative enough, these results provide an estimate of abundance for bioactive secondary metabolites produced by *Streptomyces* sp. SAJ15. Further searches based on mass features revealed that strain SAJ15 produces few known compounds, which were predicted to be derivatives of piericidin and cholyglycine compounds. This LC-MS-based secondary metabolite profiling clearly indicated that *Streptomyces* sp. SAJ15 has a potential to produce diverse compounds while culturing in appropriate media [6,8,14].

### 2.6. Integration of Metabolite Profile with BGCs

**Figure 5:**
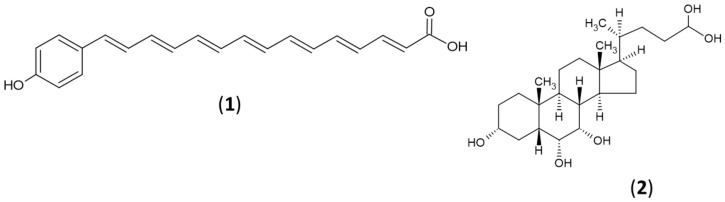
Arylpolyene (**1**) and cholyglycine (**2**) compounds detected in the *Streptomyces* sp. SAJ15.

The compounds predicted by metabolites profiling were mapped within the biosynthetic potential found in the genome of SAJ15. There were three compounds with structural features that matched with predicted products of BGCs present in the genome of SAJ15, which belongs to arylpolyene (**1**) and cholyglycine (**2**) types (Figure 5).

Piericidin-derivative: Two compounds detected at RT 12.3 and 15.9, respectively, were found to have a mass profile similar to salmeterol. Salmeterol has an aryl alkyl group with a chain length of 11 atoms from the amine [48]. Mining of the genome of *Streptomyces* sp. SAJ15 resulted in the identification of a type I PKS arylpolyene BGC. The arrangements of genes in the identified type I PKS arylpolyene BGC14 are presented in the Supplementary Information, Figure S9. Genes present in this type I PKS arylpolyene BGC14 of SAJ15 showed a 100% similarity sequence to piericidin BGC of *Streptomyces* sp. SCSIO, which produces piericidin A1 [49]. However, the PKS arylpolyene BGC of SAJ15 had two additional beta-ketoacyl synthases (Supplementary Information, Figure S10). Overall, the presence of an arylpolyene BGC in the genome of SAJ15, and detection of an arylpolyene compound in the crude-extract of this organism, strongly suggest that strain SAJ15 produces a piericidin-derivative, which may be different from the already known piericidin compounds.

Cholyglycine derivative: BGC6 responsible for the synthesis of cholyglycine-related compounds was detected through antiSMASH with a length of 26.78 Kbp. This agreed with the detection of a compound 3,6-Dihydroxycholan-24-oic acid (Supplementary Information, Figure S11) in the extract of *Streptomyces* sp. SAJ15. BGC6 consists of 19 genes, including: (i) eight biosynthetic genes, (ii) one regulator gene, and (iii) four additional genes that are probably involved in the synthesis of the compound. According to antiSMASH results, 76% of genes show a similarity to a hopene BGC. The BGC6 is predicted to direct the synthesis of a terpenoid compound through two squalene synthases and a terpene synthase. Interestingly, 3,6-Dihydroxycholan-24-oic acid has not yet been purified from any *Streptomyces*.

The advent of the modern LC-MS-based approach has allowed the concurrent detection of metabolites in complex mixtures [50]. Integration of the resulted metabolite profile with BGC promises robust insight into the biosynthesis of natural products [51]. With reference to the chemical nature/class of the detected compounds, relevant BGCs were probed using comparative genomics. This successful probing of BCGs presents a link between the chemistry and biosynthetic genes, and supports metabolome-guided exploration of biosynthetic systems of bacteria [51,52].

## 3. Materials and Methods

### 3.1. Strain and Culture Condition

Strain SAJ15, considered in the current study, was isolated previously from arid soil collected from Bikaner (East Longitude 28°1′ and North Latitude 73°19′), India. Modified inorganic salt broth (Starch 10 g, yeast extract 4 g, (NH_4_)_2_SO_4_ 2 g, K_2_HPO_4_ 1 g, MgSO_4_·7H_2_O 1 g, CaCO_3_ 1 g in 1 L distilled water) was used as growth medium for maintenance of the strain SAJ15. Spores of the strain were inoculated into 50 mL of culture medium and incubated at 30 ± 2 °C with continuous shaking at 220 rpm for 7 days.

### 3.2. 16S rDNA-Based Strain Identification

The mycelium was harvested from the fresh culture of strain SAJ15 grown in ISP2 broth [53] by centrifugation at 10,000 rpm for 3 min and washed twice with sterile Milli-Q water. Genomic DNA was extracted from the harvested mycelia using *Streptomyces* DNA isolation kit (HiMedia, Mumbai, India) according to the manufacturer’s protocol. The 16S rDNA was PCR amplified, sequenced, and processed following a previously described standard method [9]. For taxonomic identification, a matrix with related 16S rDNA sequences, including *Streptomyces* strains isolated from the desert, was constructed and aligned using MUSCLE v3.8.31 [54] with default parameters and edited with Gblocks v0.91 [55] to remove ambiguous positions. The aligned 16S rRNA gene sequences were used to reconstruct the phylogenetic tree using MrBayes v3.2 [56] with a gamma distribution type range with 1,000,000 generations. Graphical representations of the phylogenetic trees were obtained with FigTree v1.4.2 (http://tree.bio.ed.ac.uk/software/figtree/).

### 3.3. Genome Sequencing, Assembly, and Annotation

For the genome sequencing, the quantity of the DNA isolated from strain SAJ15 was examined by the PicoGreen dsDNA quantitation method (Thermo Scientific, Waltham, MA, USA) using a Victor 3 fluorometer (PerkinElmer, Waltham, MA, USA). Subsequently, a paired-end (PE) library was prepared from the quality checked DNA sample using a TruSeq DNA PCR-Free sample preparation kit (Illumina Inc., San Diego, CA, USA) according to the manufacturer’s protocol. One microgram of good quality DNA was used as a starting material for the preparation of PE library. Quality of the prepared library was validated using a DNA 1000 chip on Bioanalyzer 2100 (Agilent Technologies, Santa Clara, CA, USA). The DNA library was sequenced using the MiSeq platform with MiSeq Reagent Kit2 (2 × 100 bp; Illumina Inc., San Diego, CA, USA).

The quality of the raw Illumina reads was checked using an NGS-QC Toolkit v2.3.3 [57]. High-quality reads were considered for de novo genome assembly. Primary assembly and scaffolding were performed using Velvet v1.2.10 [58] and SSPACE v 3.0 [59], respectively. The assembled genome was validated using Bowtie v2.2.2 [60]. The rRNA and tRNA were predicted using RNAmmer [61] and ARAGORN v1.2.36 [62], respectively. The final assembled genome was annotated using RAST v2.0 [63]. No plasmid sequences were found. Draft genome assembly was further checked for the presence of plasmids using online tool PlasmidFinder v1.3 [64] and also checked for the presence of bacteriophage sequences using the PHASTER tool [65].

High-quality reads had ≥70% HQ bases (i.e., ≥20 phred score) and an average read length of 101.0 bp. The 75,933,312 high quality filtered reads covered 7,669,264,512 bp (7669.2 Mb). In the case of another desert isolate *Streptomyces* sp. Wb2n-1132, 123,881 raw reads covering 604,678,994 Mb were obtained using the PacBio Sequencing method. However, the genome of closer strains *Streptomyces* sp. SAS02 and *S. palmae* CMU-AB204^(T)^ has not been sequenced to date. Both the base and read quality distribution was analyzed in raw and quality filtered reads. The overall mean phred score was 32.5. There were no reads with an average phred quality score lower than 23. It suggested that low-quality bases were not in a subset of the reads, distributed across all raw reads, while the quality-filtered reads were free from low-quality bases and suitable for subsequent genome assembly. The quality-filtered reads were primarily assembled into 109 contigs (~0.07 Mb average length), with parameters set to have a minimum contig length ≥ 200 bp. With an average length of 75,257.95 bp, the 109 contigs spanned 8,203,117 bp (~8.2 Mb). Maximum contig length was 1,295,609 bp (~1.29 Mb). The contigs were further assembled into 70 scaffolds, with maximum link ratio ≥ 0.5 using SSPACE v 3.033 [59]. No plasmids were found in the assembled genome. A total of 11 prophage regions were identified, of which none was intact, 9 regions were incomplete, and 2 regions were questionable.

### 3.4. Genome Mining of Novel Biosynthetic Gene Clusters in SAJ15

Specialized compound genome mining of the SAJ15 genome assembly plus *Streptomyces* genome assemblies was performed using the antiSMASH 5.0 pipeline [38]. Only the complete BGCs detected by antiSMASH were included in the BGC database constructed with 192 public *Streptomyces* genome assemblies isolated from different environments conformed by 156 different species of *Streptomyces*. The public genome assemblies were obtained from the National Center for Biotechnology Information (NCBI, September 2019; Table S3). To construct the BGC sequence similarity networks of our *Streptomyces*-BGCs dataset, BiG-SCAPE pipeline was used [43]. BiG-SCAPE was run using the default parameters, including the singletons and those BGCs from the MiBIG database (https://mibig.secondarymetabolites.org/).

The difference in relative proportion of unique BGCs to the total BGCs in the *Streptomyces* of arid and non-arid environments was tested using the constructed database. Welch’s *t*-test was applied to evaluate statistical significance. Relative abundance of eight BiG-SCAPE families (GCFs) including (1) others, (2) terpenes, (3) NRPs, (4) RiPPs, (5) PKSI, (6) PKS-other, (7) PKS-NRPs hybrid, and (8) saccharides was compared between arid and non-arid environments. According to the data type, Welch’s *t*-test or Pearson chi-square tests were used for comparisons between arid and non-arid environments.

### 3.5. Determination of Growth and Metabolite Production

Strain SAJ15 was initially grown on International *Streptomyces* Project (ISP-4) agar [53] supplemented with 0.4% (*w/v*) yeast extract at 30 °C for 7 days. The fully-grown culture with spores was gently scraped with 3 mL of sterilized water added to suspend the spores into water. The spore suspension was transferred to a sterile vial, and the final volume was made to 10 mL. Then, it was vortexed vigorously to obtain homogenous spore suspension. The homogenous suspension was filtered using cotton and centrifuged for 10 min at 12,000 rpm. Spore pellets were collected and resuspended in 1 mL of sterile water. The spore suspension was inoculated into 50 mL of ISP-2 medium [53] prepared in a 200 mL Erlenmeyer flask. The inoculated flask was incubated on a shaker at 120 rpm at 30 °C for 3 days and used as a starter culture.

Strain SAJ15 was cultivated in five different production media to find the ideal one for secondary metabolite production. A 6 mL aliquot of the starter culture was then transferred to 2 L Erlenmeyer flasks containing 600 mL of five different sterile production media (Supplementary Information, Table S6) and incubated at 30 °C on a rotary shaker with 180 rpm for 10 days. Flasks containing un-inoculated media were also maintained as a control.

In order to determine the growth of *Streptomyces* sp. SAJ15 in different production media, mycelial biomass was calculated in triplicates. Mycelial pellets were collected by centrifugation of spent broth (after incubation) at 1000 rpm for 10 min. The pellets were then dried at 60 °C and weighed to determine the growth. Cell-free supernatant of each spent medium was used for the extraction of compounds. Five hundred milliliters of each supernatant were taken in a different separation funnel and mixed with an equal volume of ethyl acetate. The mixture was agitated for 10 min and left undisturbed for the separation of the organic layer. The organic layer containing secondary metabolites was separated and centrifuged at 5000 rpm for 15 min to remove traces of fermentation broth. The crude extract was dried using a rotational vacuum concentrator and re-suspended in 5 mL of HPLC grade methanol. The solution was filtered using 0.22 µm membrane filters and stored for HPLC analysis.

#### 3.5.1. HPLC-PDA Analysis

The filtered crude extracts and control extracts were analyzed on an analytical HPLC system (SHIMADZU, Kyoto, Japan) equipped with LC-20AP (pump), SPD-M20A (Photodiode Array detector), and an Enable C18 reverse-phase column (250 × 4.5 mm, 5 μm). HPLC grade water (solvent A) and methanol (solvent B) were used as solvents. The injection volume of samples was 10 µL. The samples were analyzed by linear gradient elution using 90% water as solvent A and methanol as solvent B at a flow rate of 1 mL min^−1^. The gradient applied was from 10 to 100% solvent B in 30 min with 20 min holding at 100% solvent B. Compounds were detected with a wavelength range from 200 to 600 nm. The chromatogram was recorded using Lab Solution software (SHIMADZU, Kyoto, Japan). Chromatograms of different crude extracts were compared, and peaks were noted to represent the number of compounds produced by *Streptomyces* sp. SAJ15 in different media. The data comparison module of Lab solution software (SHIMADZU, Japan) was used for the HPLC data analysis. The crude extract found to have the maximum number of compounds was considered for metabolomics analysis using High-Resolution Liquid Chromatography (HR-LC-MS) coupled with mass spectrometry.

#### 3.5.2. HR-LC-MS Analysis

A selected crude extract was analyzed using an HR-LC-MS comprising a 1290 Infinity UHPLC system coupled to 6550 iFunnel Q-TOFs (Agilent Technologies, Santa Clara, CA, USA). The chromatographic separation was achieved using a Phenomenex Gemini-NX C18 HPLC column (150 mm × 2.0 mm, 3 µm, Phenomenex, Torrance, CA, USA) with a mobile phase that consisted of HPLC-grade water (A) and acetonitrile (B) added with 0.1% formic acid in a linear gradient. Mobile phase flow rate was set at 0.2 µL min^−1^. The sample injection volume was 20 μL. Electrospray Ionization (ESI) was used to ionize and detect compounds after chromatographic separation. Acquisition parameters were as follows: minimum *m/z* 130, maximum *m/z* 1000, and scan rate 1 s^−1^. Ionizing source parameters were as follows: gas temperature 250 °C, gas flow 13 min^−1^, nebulizer pressure 30 psi, sheath gas temperature 300 °C, and sheath gas flow 13 min^−1^. Scan source parameters were as follows: Capillary voltage 3500 V, nozzle voltage 1000 V, fragmentor voltage 175 V, skimmer1 voltage 65.0 V, and octopole RF Peak voltage 750 V. Data recording and processing were performed using Agilent MassHunter software (6200 series TOF, version B.05.01, Agilent Technologies, USA). Metabolite peaks were assigned by searching their accurate mass, referencing the compounds database provided with the Agilent UHPLC-MS-Q-TOF system. The search parameters set were as follows: mass tolerance (accurate mass) ≤ 5 ppm; a maximum number of peaks to search when peaks are not specified graphically 5; charge carriers H^+^, K^+^, and Na^+^.

#### 3.5.3. HR-LC-MS Data Mapping to BGCs of Strain SAJ15

Compounds identified from the UHPLC-MS-Q-TOF data were mapped to BGCs present in the genome of *Streptomyces* sp. SAJ15 to establish the relationship between them. Despite the limitations in mapping the compounds to BGCs due to post-transcriptional modifications, putative relationships were established based on the enzymes predicted in the BGCs. Prediction of the chemical structures of the putative specialized metabolites associated with unique biosynthetic gene clusters was performed after domain identification and specificity prediction, mainly of adenylation and acyl transfer domains, with antiSMASH [38] and Polyketide synthases/non-ribosomal peptide synthetase (PKs/NRPs) analysis [66].

## 4. Conclusions

*Streptomyces* spp. have a long-term reputation in natural product research due to their unprecedented capacity to produce diverse compounds. Therefore, we extensively studied the strain SAJ15 previously isolated from desert soil. Draft genome of *Streptomyces* sp. SAJ15 was obtained in 69 contigs with a total genome size of 8.2 Mb and 72.54% G + C content. Interestingly, genome mining by antiSMASH for BGCs that encode secondary metabolites revealed that a total of 36 complete BGCs are present in the *Streptomyces* sp. SAJ15 genome. Out of them, 75% were putatively novel (with sequence identity cutoff <20%), which implies the scope of strain SAJ15 in the field of natural product research. Integration of the HR-LC-MS-based metabolite profile with BGCs found in the SAJ15 genome suggested the production of arylpolyene and cholyglycine compounds. Moreover, our results support the hypothesis that *Streptomyces* species of desert environments have evolved with unique biosynthetic potential. Profiling of phylogenetic relationships revealed that *Streptomyces* strains of desert origin are closer to each other and relatively distinct from *Streptomyces* of other environments. Relative abundance of different BGC classes in arid *Streptomyces* differed from non-arid counterparts, which suggested niche specific evolution of biosynthetic capabilities in *Streptomyces*.

## Data Availability

The data and analyses from the current study are available from the corresponding authors upon reasonable request. Sequence data has been deposited in the GenBank under accession number NISY00000000.

## Supplementary Materials

The following are available online, Table S1: *Streptomyces* strains used to re-construct the 16S rRNA phylogenetic tree, Table S2: List of BGCs found in the genome of *Streptomyces* sp. SAJ15 by antiSMASH, Table S3: *Streptomyces* genomes used to construct the *Streptomyces*-BGCs dataset, Table S4: Growth (biomass) and secondary metabolites (crude extract) of strain SAJ15 cultivated in different production media, Table S5: RT and mass features of putatively novel hits that are dissimilar to known natural compounds, Table S6: Composition of different production media used for secondary metabolites production by SAJ15, Figure S1: BiG-SCAPE family classified as Others. BGC sequence similarity networks of BGCs from 179 Streptomyces genomes, Figure S2: BiG-SCAPE family classified as NRPs, Figure S3: BiG-SCAPE family classified as PKS-NRPs hybrid, Figure S4: BiG-SCAPE family classified as PKSI, Figure S5: BiG-SCAPE family classified as PKS-Other, Figure S6: BiG-SCAPE family classified as RiPPs, Figure S7: BiG-SCAPE family classified as Terpenes, Figure S8: UHPLC-MS-Q-TOF chromatogram of culture extract (CE15-PM01) of *Streptomyces* sp. SAJ15, Figure S9: Putative relationship between the BGC14 and salmeterol derivatives detected by UHPLC-MS-Q-TOF, Figure S10: Hybrid PKs-arylpolyene BGC of SAJ15 had two additional beta-ketoacyl synthase compared to piericidin A BGC, Figure S11: Putative relationship between the BGC_06 and cholyglycine related compound (hydroxycholan-24-oic acid) detected by UHPLC-MS-Q-TOF.
