# Supplementary material for: Desert Environments Facilitate Unique Evolution of Biosynthetic Potential in Streptomyces
Source: Molecules. 2021 Jan 22;26(3):588. doi: 10.3390/molecules26030588 (PMC7865587; doi:10.3390/molecules26030588)
Supplement: Supplementary file 1 [file molecules-26-00588-s001.zip › Figures S1-S11_v2.pdf]

Supplementary information figures

Desert Environments Facilitate Unique Evolution of Biosynthetic Potential in *Streptomyces*

Kunjukrishnan Kamalakshi Sivakala [1,2,†], Gutiérrez-García Karina [3,4,†], Polpass Arul Jose [2,5,*], Thangadurai Thinesh [6], Rangasamy Anandham [5], Barona-Gómez Francisco [3] and Natesan Sivakumar [1,*]

[1] School of Biotechnology, Madurai Kamaraj University, Madurai 625021, India; sivakalaarul@gmail.com (K.K.S.); siva.biotech@mkuniversity.org (N.S.)

[2] Robert H. Smith Faculty of Agriculture, Food and Environment, Hebrew University of Jerusalem, Rehovot 7610001, Israel

[3] Evolution of Metabolic Diversity Laboratory, Unidad de Genómica Avanzada (Langebio), Cinvestav-IPN, Km 9.6 Libramiento Norte, Carretera Irapuato-León, CP 36821, Irapuato, Guanajuato, Mexico; kgutierrez@carnegiescience.edu (G.-G.K.); francisco.barona@cinvestav.mx (B.-G.F.)

[4] Department of Embryology, Carnegie Institution for Science, Baltimore, MD 21218, USA

[5] Department of Agricultural Microbiology, Agricultural College and Research Institute, Tamil Nadu Agricultural University, Madurai 625104, India; anandham@tnau.ac.in (R.A.); arulmku@gmail.com (P.A.J.)

[6] Department of Microbiology, School of Life Sciences, Pondicherry University, Puducherry 605014, India; thina.sathesh@gmail.com

* Authors to whom correspondence should be addressed.

† These authors contributed equally to this work

Figure S1

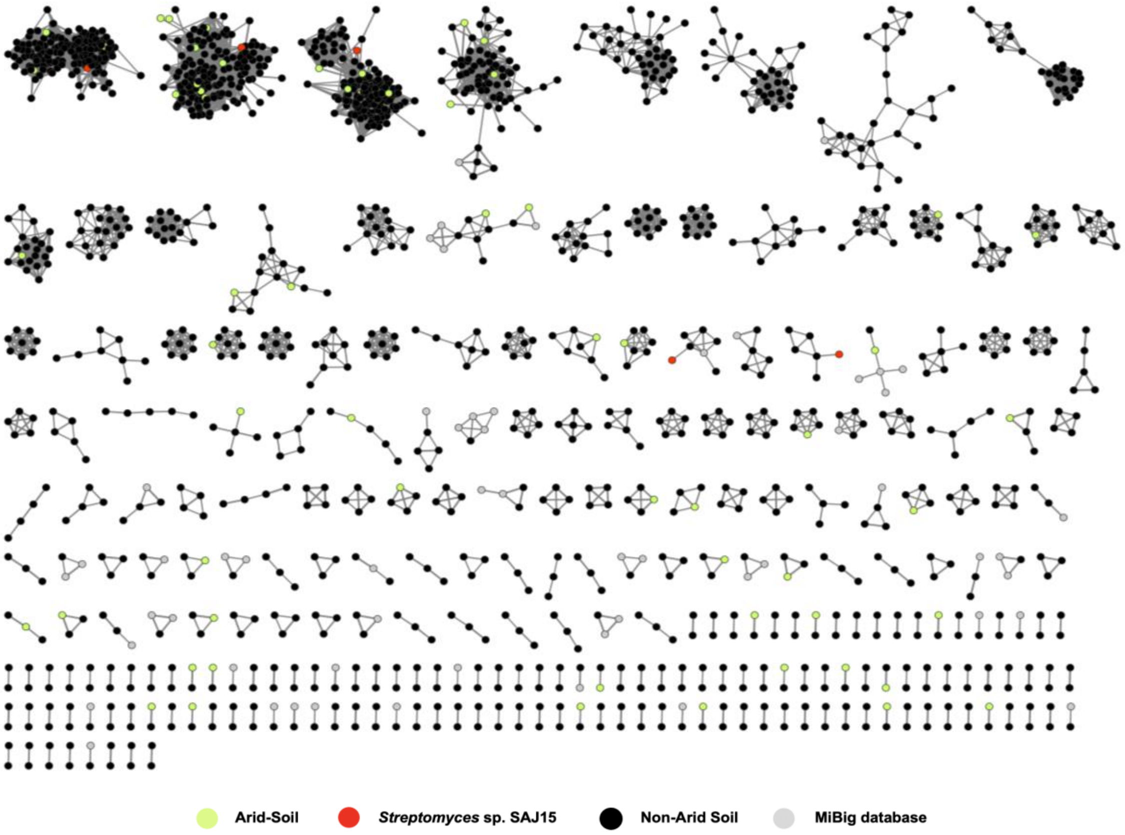

Figure S1. BiG-SCAPE family classified as Others. BGC sequence similarity networks of BGCs from 179 *Streptomyces* genomes. 1,540 *Streptomyces*-BGCs were classified into this GCF.

Figure S2

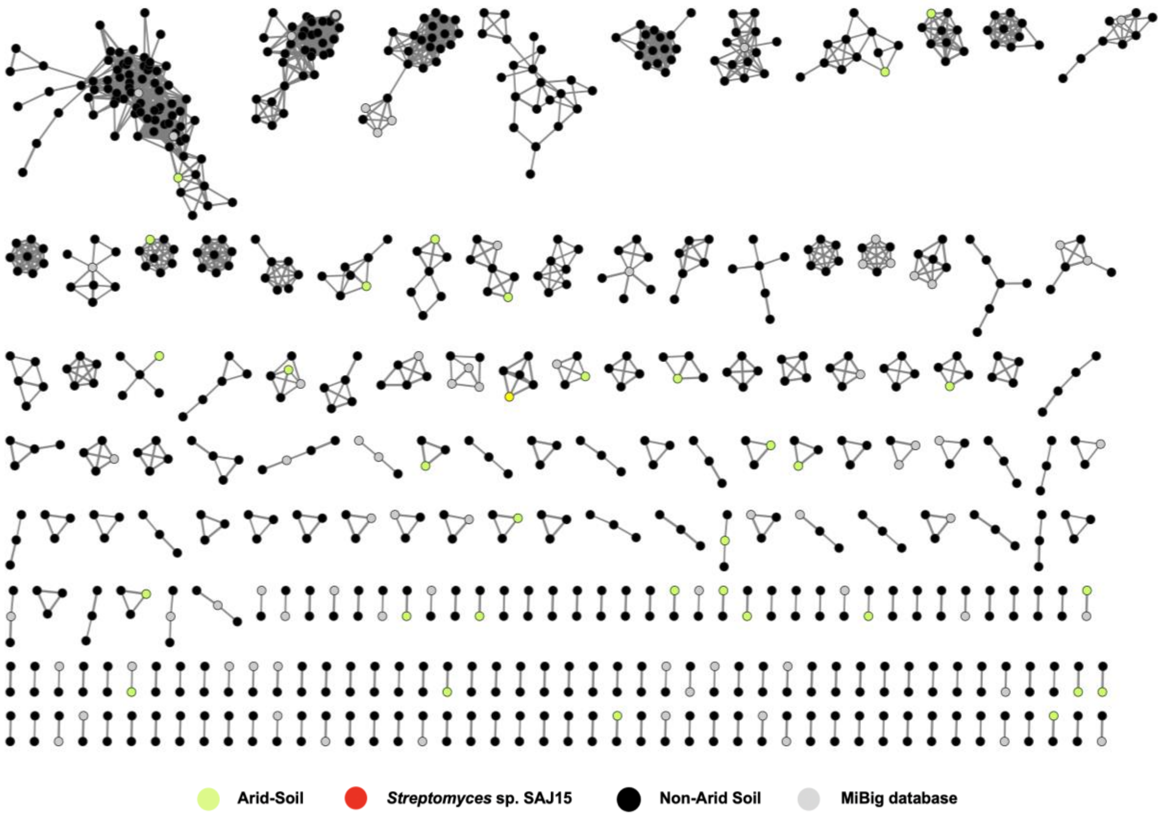

Figure S2 BiG-SCAPE family classified as NRPs. BGC sequence similarity networks of BGCs from 179 *Streptomyces* genomes. 761 *Streptomyces*-BGCs were classified into this GCF.

Figure S3

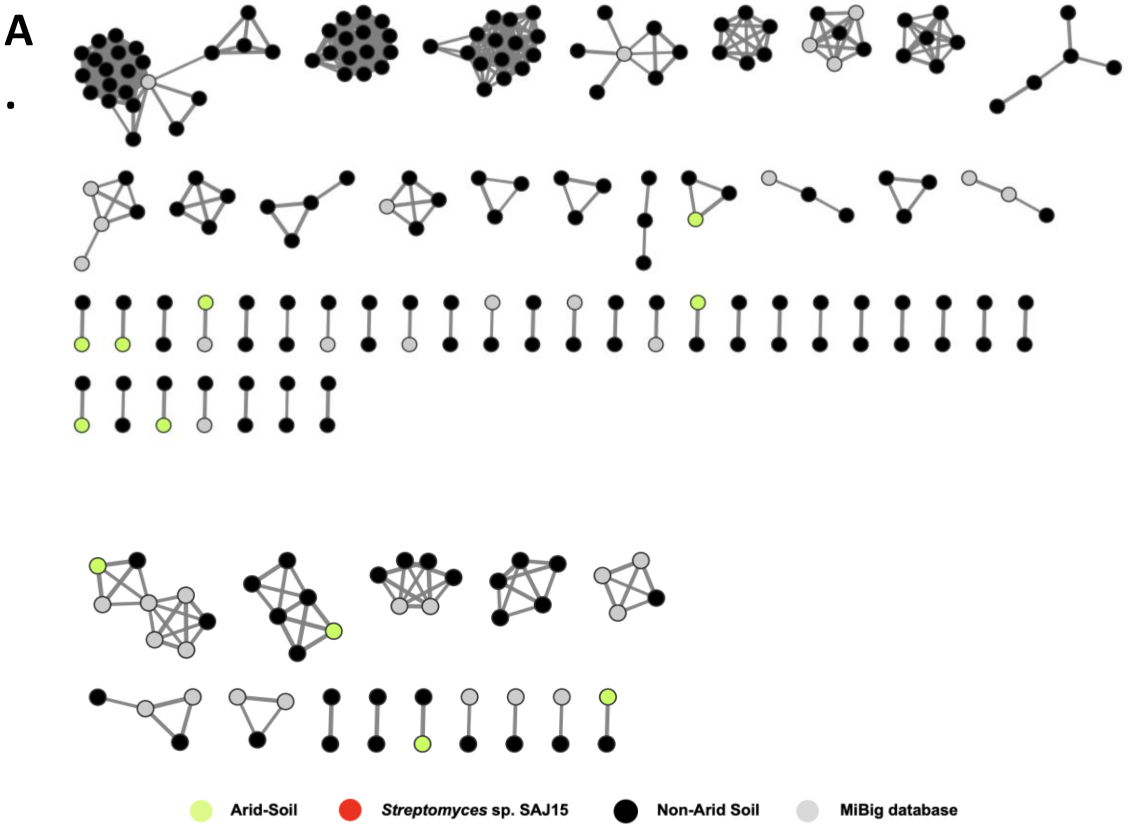

Figure S3. A. BiG-SCAPE family classified as PKS-NRPs hybrid. BGC sequence similarity networks of BGCs from 179 *Streptomyces genomes*. 164 *Streptomyces*-BGCs were classified into this GCF. B. BiG-SCAPE family classified as Saccharides. 33 *Streptomyces*-BGCs were classified into this GCF

Figure S4

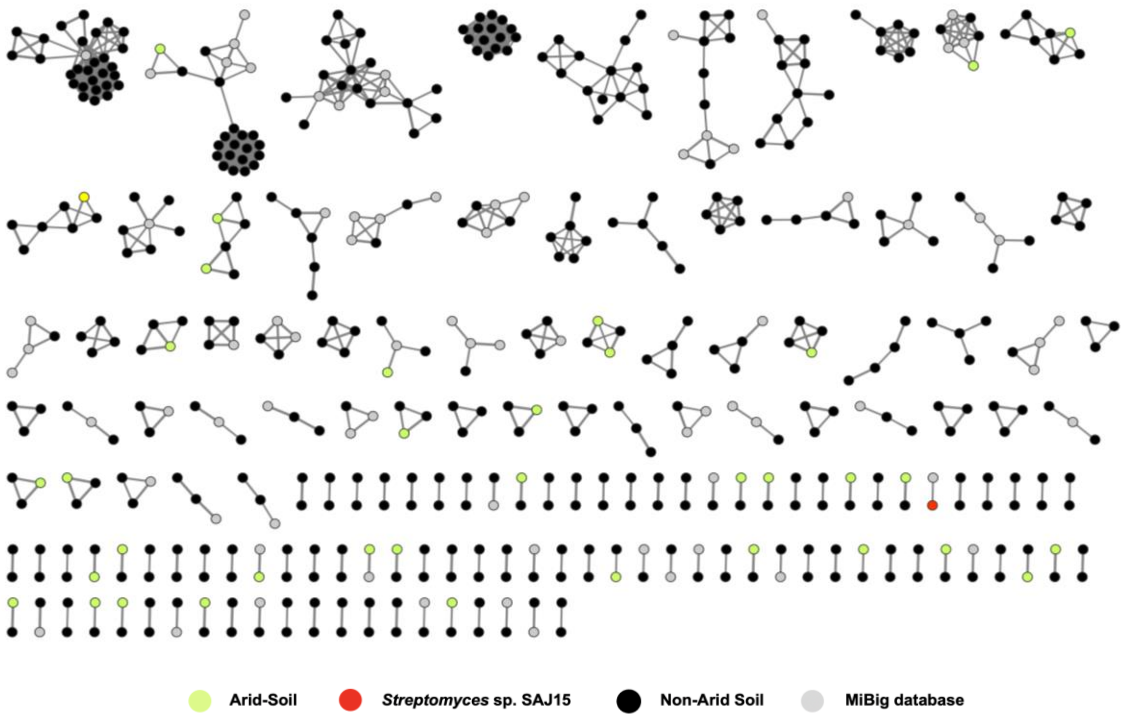

Figure S4. BiG-SCAPE family classified as PKSI. BGC sequence similarity networks of BGCs from 179 *Streptomyces* genomes. 457 *Streptomyces*-BGCs were classified into this GCF.

Figure S5

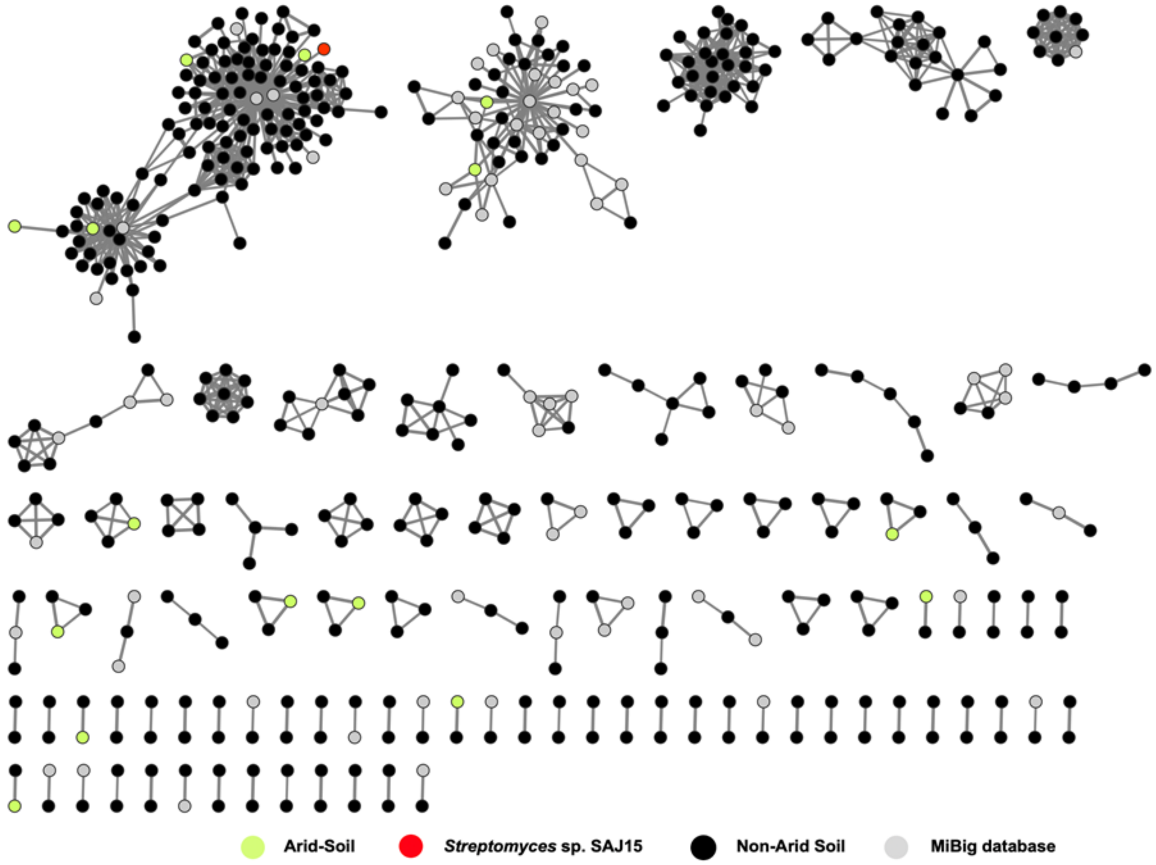

Figure S5. BiG-SCAPE family classified as PKS-Other. BGC sequence similarity networks of BGCs from 179 *Streptomyces* genomes. 423 *Streptomyces*-BGCs were classified into this GCF.

Figure S6

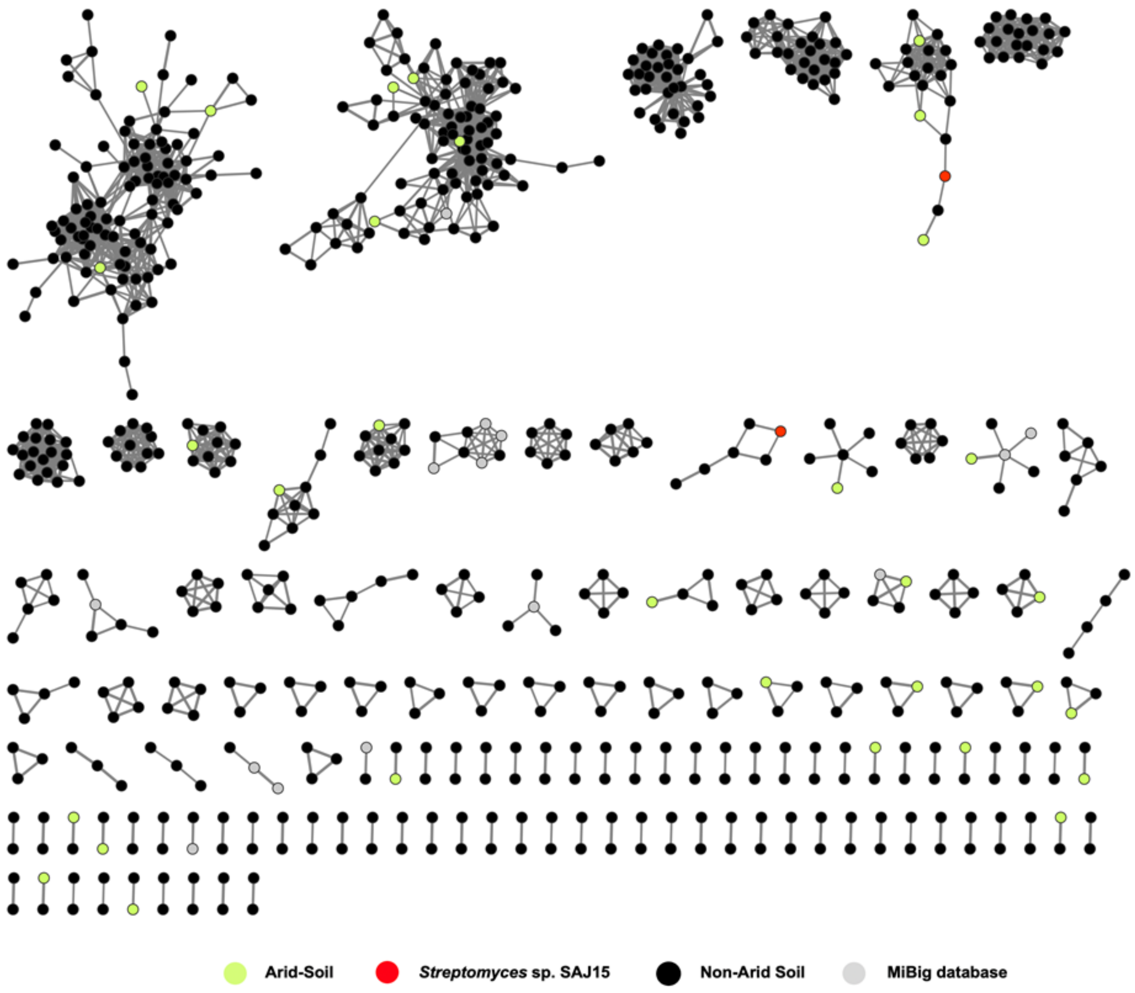

Figure S6. BiG-SCAPE family classified as RiPPs. BGC sequence similarity networks of BGCs from 179 *Streptomyces* genomes. 654 *Streptomyces*-BGCs were classified into this GCF.

Figure S7

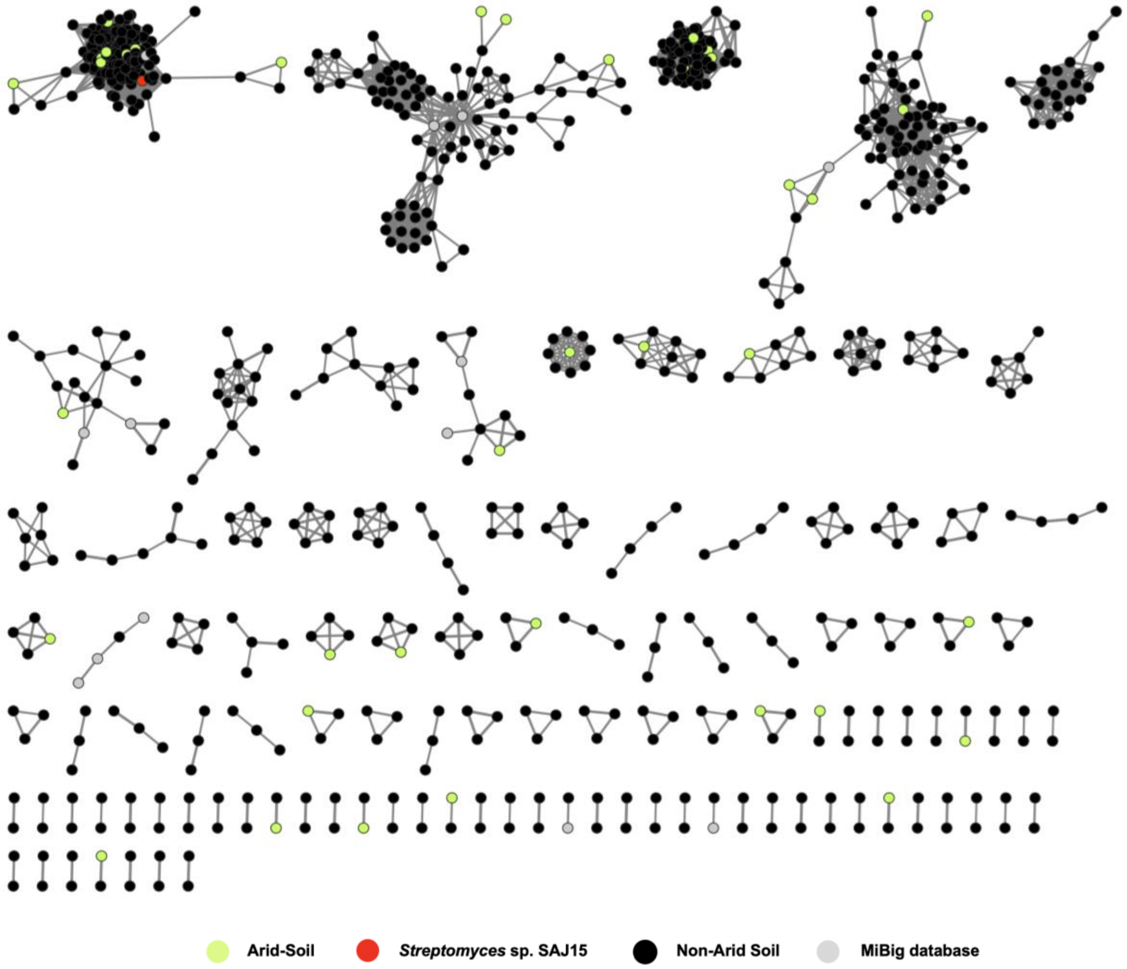

Figure S7. BiG-SCAPE family classified as Terpenes. BGC sequence similarity networks of BGCs from 179 *Streptomyces* genomes. 799 *Streptomyces*-BGCs were classified into this GCF.

Figure S8

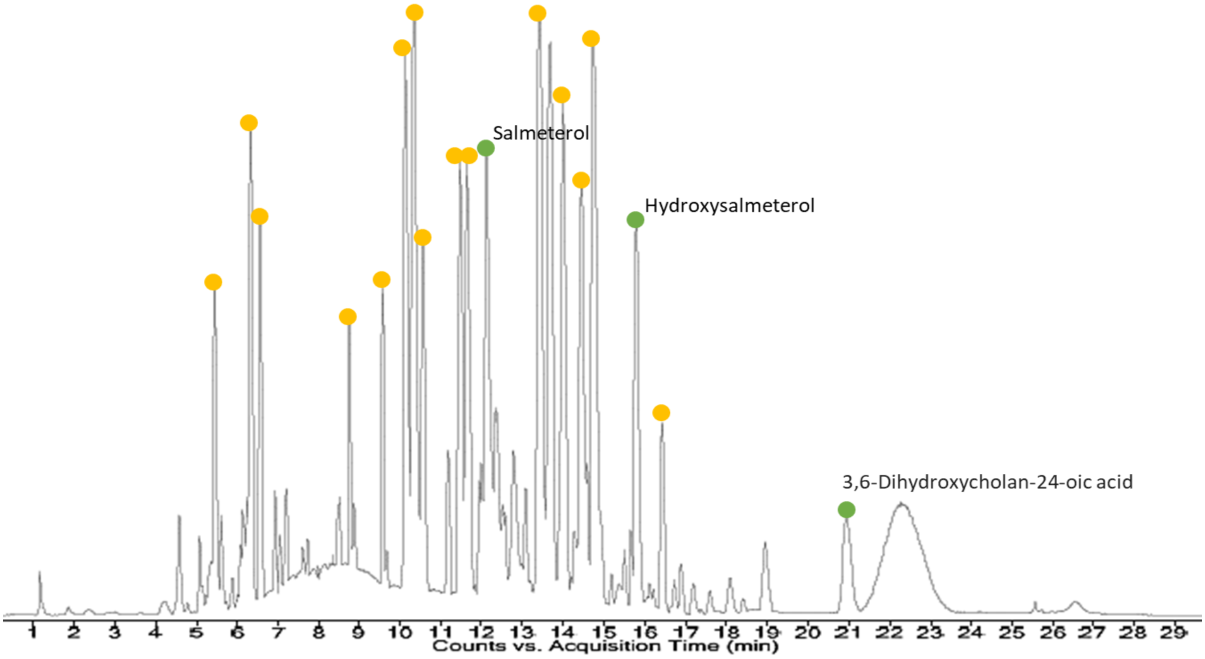

Figure S8: UHPLC-MS-Q-TOF chromatogram of culture extract (CE15-PM01) of *Streptomyces* sp. SAJ15. Peaks marked with green corresponds predicted to be known compounds, while yellow corresponds putatively new compounds (didn't match with mass features of known compounds).

Figure S9

a)

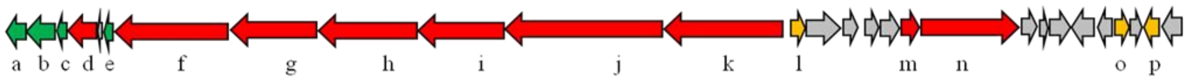

b)

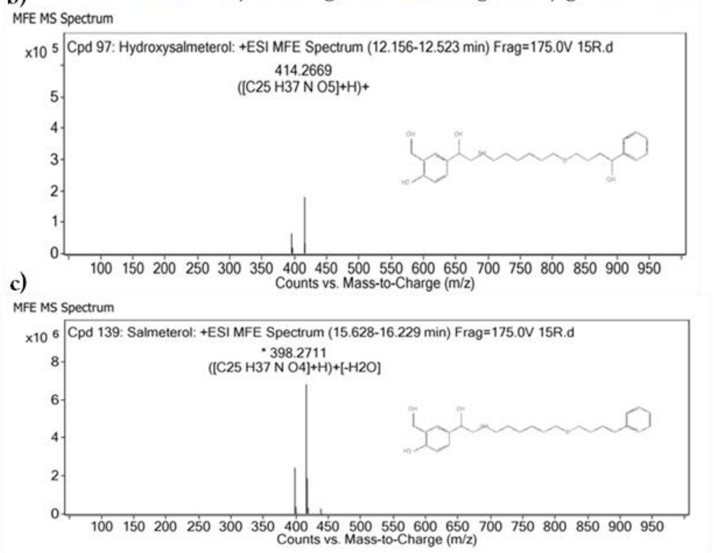

d)

Gene ID	Function / Protein
a	FAD linked oxidase domain protein
b	Monooxigenase
c	Methyltransferase
d	Asparagine synthase
e	Methyltransferase
f	Beta-ketoacyl synthase
g	Beta-ketoacyl synthase
h	Beta-ketoacyl synthase
i	Beta-ketoacyl synthase
j	Beta-ketoacyl synthase
k	Malonil-CoA acyl carrier protein transacylase
l	Transcriptional regulator
m	Beta-ketoacyl shyntase
n	LysR family transcriptional regulator
o	RNA polymerase 24 subunit

Figure S9: Putative relationship between the BGC14 and salmeterol derivatives detected by UHPLC-MS-Q-TOF; arrangements of genes in the identified I pks - arylpolyene BGC (a), structure of hydroxysalmeterol (b) and salmeterol (c), and identified functions/proteins of genes found in type I pks - arylpolyene BGC (d).

Figure S10

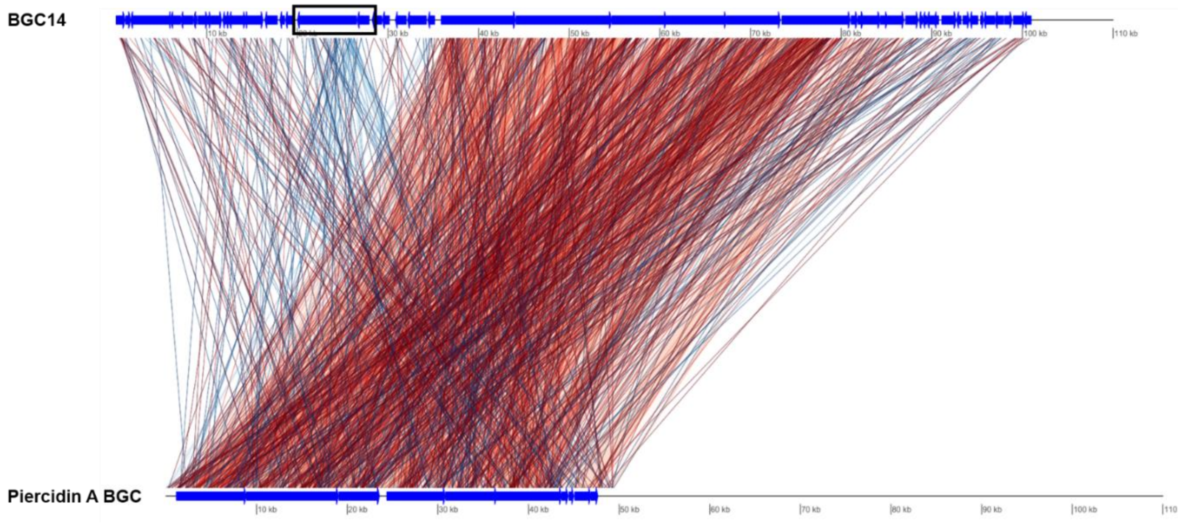

Figure S10: Hybrid PKs - arylpolyene BGC of SAJ15 had two additional beta-ketoacyl synthase compared to piericidin A BGC.

Figure S11

a)

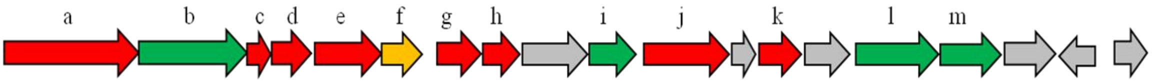

b)

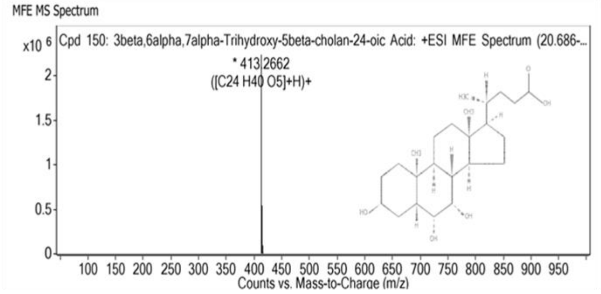

c

Gene ID	Function / Protein
a	Short-chain dehydrogenase/reductase SRD
b	Short-chain dehydrogenase/reductase SRD
c	Hipotetical protein
d	Short-chain dehydrogenase/reductase SRD
e	Cytochrome P450
f	TetR family transcriptional regulator
g	squalene synthase
h	squalene synthase
i	Polyprenyl synthetase
j	Terpene cyclase
k	hopanoid biosynthesis associated radical SAM protein
l	1-Deoxy-D-xylulose-5-phosphate synthase
m	Aminotransferase class III

Figure S11: Putative relationship between the BGC_06 and cholyglycine related compound (hydroxycholan-24-oic acid) detected by UHPLC-MS-Q-TOF. Arrangements of genes in the identified BGC_06 (a), structure of 3,6-Dihydroxycholan-24-oic acid (b), and identified functions/proteins of genes found in BGC_06 (c).
